# Rapid induction of orthotopic hepatocellular carcinoma in immune-competent rats by non-invasive ultrasound-guided cells implantation

**DOI:** 10.1186/1471-230X-10-83

**Published:** 2010-07-22

**Authors:** Hoi-Hung Chan, Tian-Huei Chu, Hsin-Fan Chien, Cheuk-Kwan Sun, E-Ming Wang, Huay-Ben Pan, Hsiao-Mei Kuo, Tsung-Hui Hu, Kwok-Hung Lai, Jiin-Tsuey Cheng, Ming-Hong Tai

**Affiliations:** 1 Department of Biological Sciences, National Sun Yat-sen University, 70 Lien-Hai Road, Kaohsiung 80424, Taiwan; 2 Division of Gastroenterology, Department of Internal Medicine, Kaohsiung Veterans General Hospital, 386 Ta-Chung 1st Road, Kaohsiung 81362, Taiwan; 3 Department of Education and Research, Kaohsiung Veterans General Hospital, 386 Ta-Chung 1st Road, Kaohsiung 81362, Taiwan; 4 Department of Radiology, Kaohsiung Veterans General Hospital, 386 Ta-Chung 1st Road, Kaohsiung 81362, Taiwan; 5 Institute of Biomedical Sciences, National Sun Yat-sen University, 70 Lien-Hai Road, Kaohsiung, 80424, Taiwan; 6 Division of General Surgery, Department of Surgery, Chang Gung Memorial Hospital - Kaohsiung Medical Center, Chang Gung University College of Medicine, 123 Ta-Pei Road, Niao-Sung Hsiang, Kaohsiung Hsien 83304, Taiwan; 7 School of Medicine, National Yang-Ming University, No 155, Sec 2, Li-Nong Street, Pei-Tou, Taipei 112, Taiwan; 8 Division of Hepato-Gastroenterology, Department of Internal Medicine, Chang Gung Memorial Hospital - Kaohsiung Medical Center, Chang Gung University College of Medicine, 123 Ta-Pei Road, Niao-Sung Hsiang, Kaohsiung Hsien 83304, Taiwan

## Abstract

**Background:**

The fact that prognoses remain poor in patients with advanced hepatocellular carcinoma highlights the demand for suitable animal models to facilitate the development of anti-cancer medications. This study employed a relatively non-invasive approach to establish an orthotopic hepatocellular carcinoma model in immune-competent rats. This was done by ultrasound-guided implantation of cancer cells and the model was used to evaluate the therapeutic efficacy of short-term and low-dose epirubicin chemotherapy.

**Methods:**

Rat Novikoff hepatoma cells were injected percutaneously into the liver lobes of Sprague-Dawley rats under the guidance of high resolution ultrasound. The implantation rate and the correlation between dissected and ultrasound-measured tumor sizes were evaluated. A similar induction procedure was performed by means of laparotomy in a different group of rats. Pairs of tumor measurement were compared by ultrasound and computerized tomography scan. Rats with a successful establishment of the tumor were divided into the treatment (7-day low-dose epirubicin) group and the control group. The tumor sizes were non-invasively monitored by the same ultrasound machine. Blood and tumor tissues from tumor-bearing rats were examined by biochemical and histological analysis respectively.

**Results:**

Ultrasound-guided implantation of Novikoff hepatoma cells led to the formation of orthotopic hepatocellular carcinoma in 60.4% (55/91) of the Sprague-Dawley rats. Moreover, tumor sizes measured by ultrasound significantly correlated with those measured by calipers after sacrificing the animals (*P *< 0.00001). The rate of tumor induction by ultrasound-guided implantation was comparable to that of laparotomy (55/91, 60.4% vs. 39/52, 75%) and no significant difference in sizes of tumor was noted between the two groups. There was a significant correlation in tumor size measurement by ultrasound and computerized tomography scan. In tumor-bearing rats, short-term and low-dose epirubicin chemotherapy caused a significant reduction in tumor growth, and was found to be associated with enhanced apoptosis and attenuated proliferation as well as a decrease in the microvessel density in tumors.

**Conclusions:**

Ultrasound-guided implantation of Novikoff hepatoma cells is an effective means of establishing orthotopic hepatocellular carcinoma in Sprague-Dawley rats. Short-term and low-dose epirubicin chemotherapy had perturbed tumor progression by inducing apoptosis and neovascularization blockade.

## Background

Hepatocellular carcinoma (HCC) is the most common primary malignancy of the liver (70-85%). It is also one of the most frequent malignancies worldwide, particularly in Asia and Africa. The incidence is still rising in some countries such as Central Europe, North America and Oceania for unknown reasons [1]. Unfortunately, most of the HCC patients have non-specific symptoms [2] and will probably miss the chance of receiving curative treatment. Ultrasound (with or without contrast agents) is sensitive in detecting small HCCs while new generation computerized tomography (CT) with spiral and triphasic scanners can improve the specificity in differentiating HCC from other kinds of liver tumors. Serum α-fetoprotein (AFP) is probably the most frequently used tumor marker for the diagnosis of HCC. However, the sensitivity and specificity of AFP need further validation such as exploration of its subtypes. Routine use of percutaneous needle biopsy of HCC is controversial because of the risk of needle-track seeding and is better reserved for situations where definite histological diagnosis is mandatory [3,4]. Although tumor resection and liver transplantation are currently the mainstays of curative therapies for HCC, only 10-15% of newly diagnosed patients in Asia have resectable tumors. Local therapies such as radiofrequency ablation and alcohol injection are alternatives for small tumors and patients unsuitable for surgical intervention with comparable success rates. Transarterial chemoembolisation (TACE) is recommended for selected cases of locally advanced large unresectable tumors with good liver functional reserve and no vascular involvement [5].

Since prognoses are dismal for advanced or metastatic tumors [6], the development of a suitable model for testing new treatment modalities for HCC is urgently required. Screening of drug candidates for HCC is usually performed using xenografted HCC in immune-deficient mice such as nude or severe combined immunodeficiency (SCID) mice. In such xenografted models, tumors are relatively vulnerable because they are not grown in vascularized livers. In addition, those studies fail to delineate the efficacy of therapeutic agents in animals with intact immune systems. In order to develop clinically applicable intervention strategies for HCC, it is essential to create an immune-competent animal model bearing orthotopic HCC.

To create animal models with orthotopic HCC, the implantation of hepatoma cells through laparotomy into syngeneic or immune-deficient animals have been employed in previous studies [7,8]. However, it is rather time-consuming and traumatic. Besides, the experimental animals often suffer from adverse events such as bleeding, infection, and tumor adhesion to the tissues and organs. Conditions are even more complicated when repeated monitoring of the tumor status is needed. Percutaneous therapies such as radiofrequency ablation with the guidance of real-time Ultrasound (US) have been widely used for the treatment of HCC with high efficacy and safety [9]. An US machine can also be used as a tool for tumor implantation and measuring the subsequent changes of the created tumors [8]. However, due to the small size of rat livers compared with those of humans, it is a prerequisite to have a high resolution US machine as well as a high frequency probe for precise tumor measurement and US-guided injection. In the current study, therefore, we investigated the feasibility and efficacy of creating orthotopic HCC (transplantable liver cancer) in a rodent model by US-guided implantation. We employed Novikoff hepatoma (N1-S1) cells, which were derived from Sprague-Dawley (SD) rats administered with *N*-2-fluorenylphthalamic acid (FPA), for US-guided implantation into the liver lobes of SD rats.

Epirubicin, which is similar to doxorubicin as a derivative of anthracycline, can inhibit topoiosomerase II-α (TOP2A) enzyme through preventing the cleavage of supercoiled DNA and blocking DNA transcription and replication. It has been used both alone [10] and in combination with other anticancer agents [11,12] in the treatment of advanced HCC. Studies have shown that regimens containing either epirubicin or doxorubicin resulted in similar response rates and survival. However, epirubicin caused less bone marrow suppression and cardiotoxicity than doxorubicin [13]. Metronomic dosing, a new concept of chemotherapy first described by Browder *et al*., [14] and Klement *et al*., [15] is the use of low-dose cytotoxic drugs, either by continuous infusion or frequent administration without extended resting periods in the treatment of malignancy. It is not as toxic as the traditional maximum tolerated dose (MTD) of chemotherapy and better accepted by patients. It also has the additional benefit of targeting the tumor endothelium instead of tumor cells resulting in an anti-angiogenic effect [16].

In the current study, we first evaluated the feasibility of using US-guided implantation of N1-S1 cells to generate orthotopic HCC in rats and the reliability of using the same machine to monitor tumor progression within the rats. Subsequently, rats bearing established HCC were treated with short-term (7-day) and low-dose epirubicin chemotherapy. We aimed at evaluating the therapeutic efficacy of this method and the related mechanism underlying the treatment in this novel HCC model.

## Methods

### Cell cultures

Rat Novikoff hepatoma (N1-S1) cells were cultured in the RPMI 1640 medium (Life Technologies, Rockville, MD) containing 100 U/mL penicillin (Hyclone, Bio-Check Laboratories Ltd., USA), 100 μg/mL streptomycin (Hyclone, Bio-Check Laboratories Ltd., USA), 5% fetal calf serum (GIBCO BRL, Rockville, MD) and 2 mmol/L glutamine (Hyclone, Bio-Check Laboratories Ltd., USA) under humidified conditions in 95% air and 5% CO_2 _at 37°C. A rat hepatocyte cell line (Clone-9) derived from normal Sprague-Dawley rat liver was purchased from Bioresource Collection and Research Center ( no. 60201; Hsin-Chu, Taiwan).

### Animal studies

All experimental procedures were reviewed and approved by the Institutional Animal Care and Use Committee before the study began, and we ensured that all animals received humane care and that study protocols complied with the institution's guidelines (see Additional file 1). Male Sprague-Dawley (SD; 150 ± 50 g) rats purchased from the National Animal Center (Taipei, Taiwan) were used in this study. Guidelines for pain experiments on conscious animals were adhered to throughout the experiments. The animals were housed two per cage, in [beta]-chip lined metal cages in a central animal care facility with a 12-hour light and 12-hour dark cycle. They were fed with rat chow and water ad libitum. After anesthetization, N1-S1 cells (5 × 10^6 ^cells suspended in 100 μl of RPMI1640) were injected percutaneously into the liver parenchyma of the SD rats using a 29-gauge syringe under the guidance of real-time US with a high frequency linear array transducer operating at 7-14 MHz (GE Logiq 9^® ^Ultrasound System). Similar induction procedure was performed by means of laparotomy in a different group of rats.

Ten days after induction, the animals were anesthetized and tumor sizes were measured by US in two perpendicular diameters and expressed as the mean of the two measurements [8]. Subsequently, the rats were sacrificed and the HCC sizes were measured by calipers. Alternatively, tumor volume was estimated by the formula: volume = (shortest diameter)^2 ^× (longest diameter) × 0.5 [17]. Plasma samples were collected from tumor-bearing rats and subjected to biochemical analysis including glutamic oxaloacetic transaminase (GOT) and glutamic pyruvic transaminase (GPT) activities using an automatic biochemical analyzer (DAX96, Bayer Corp. Diagnostic, Milan, Italy). Fifteen pairs of tumor measurement were performed using US and functional (perfusion) computerized tomography (CT) scan as previously described by Kan Z [18], while the results of biochemical measurements were plotted and compared by Pearson's correlation.

### Short-term (7-day) and low-dose epirubicin chemotherapy for treatment of HCC in SD rats

After successful implantation of orthotopic HCC in the 10^th ^day, SD rats with comparable sizes of tumor were equally divided into the treatment (n = 11) group and the control (n = 11) group. Low-dose epirubicin at 0.3 mg/day/rat was injected through the tail vein in the treatment group for seven days (the risk of congestive heart failure might occur at cumulative doses greater than 900 mg m^-2^, about 300 mg kg^-1^) [19,20]. After completion of treatment, tumor sizes measured by US were compared between groups and with those before treatment. Moreover, actual tumor sizes were obtained by caliper measurement after sacrificing the animals and the tumors were also weighted. Peripheral blood was drawn from the tail veins of the rats after completion of treatment to check the white and red blood cells as well as the platelet counts.

### Western blot analysis

The protein extracts were isolated using RIPA buffer (150 mM NaCl, 50 mM HEPES pH 7, 1% Triton X-100, 10% glycerol, 1.5 mM MgCl_2_, 1 mM EGTA) containing a protease inhibitor (Roche Applied Science; Indianapolis, IN). After separation in 12.5% SDS-PAGE, protein samples were transferred onto polyvinylidene fluoride (PVDF) membrane using blotting apparatus. The membrane was blocked with 5% milk in TBS-T for 1 h. Then, it was incubated with TOP2A antibody (1:500, cell-signaling) for 1 h at room temperature. After incubation with HRP-conjugated secondary antibody (1:5000 dilutions in 5% milk) for 60 minutes, the signals on membrane were detected using ECL-plus luminol solution (Pharmacia; Piscataway, NJ) and exposed to X-ray film for autoradiogram.

### Immunohistochemical analysis

The paraffin-embedded tissue blocks were sectioned into 3-millimeter slices and mounted on poly-L-lysine-coated slides. After deparaffinization, the slides were blocked with 3% hydrogen peroxide for 10 minutes and subjected to antigen retrieval by microwave in 10 mM citrate buffer for 15 minutes. TOP2A (1:50, cell-signaling), Ki-67 antibodies (1:100 dilution, Dako, Denmark) and PECAM-1 (platelet endothelial cell adhesion molecule-1, 1:50 dilution; Santa Cruz Biotechnology Inc.) were applied onto the sections, which were then incubated at room temperature for 60 minutes followed by repeated washing with phosphate-buffered saline (PBS). Horseradish peroxidase/Fab polymer conjugate (Polymer Detection System, Zymed, USA) was then applied to the sections and the sections were incubated for 30 minutes. After rinsing with PBS, the sections were incubated with peroxidase substrate diaminobenzidine (1:20 dilution, Zymed) for 5 minutes. Thereafter, the sections were counterstained with Gill's hematoxylin for 20 seconds, dehydrated with serial ethyl alcohol, cleared with xylene, and finally mounted. For comparison, results of the percentage of Ki-67-positive staining were counted under low power fields while PECAM-1 was presented as the amount of positive staining under high power fields.

### The terminal deoxynucleotidyl transferase-mediated dUTP nick end-labeling (TUNEL) staining

The TUNEL assay was used to detect DNA fragmentation. Briefly, the hepatoma sections on slides were deparaffinized, and then washed with PBS. TUNEL analysis was performed using the *in situ *Cell Death Detection Kit Fluorescein (Roche Molecular Biochemicals; Indianapolis, IN) according to the manufacturer's protocol. TUNEL-positive cells were visualized by immunofluorescent microscopy and counted using a 20× objective. TUNEL-positive cells containing FITC were identified by co-localization with 4,6-diamidino-2-phenylindole (DAPI) staining and by morphology. More than 100 cells were counted for each variable per experiment. The slides were viewed under a fluorescence microscope with green fluorescence set at 520 nm. The cells stained green indicated apoptotic cells. For comparison, the amount of positive staining was counted under low power fields.

### Statistical analysis

The induction rate of HCC by the US-guided method was presented as the percentage of rats with successful tumor induction divided by the total number of rats enrolled. The tumor sizes were expressed as mean ± standard deviation. Pearson's correlation was applied to compare the results of US with the corresponding measurements. Continuous variables were compared with the Student's *t *test. A *P *< 0.05 on two-tailed testing was considered significant.

## Results

### Ultrasound-guided implantation of N1-S1 cells effectively induced orthotopic HCC in SD rats

**Figure 1:**
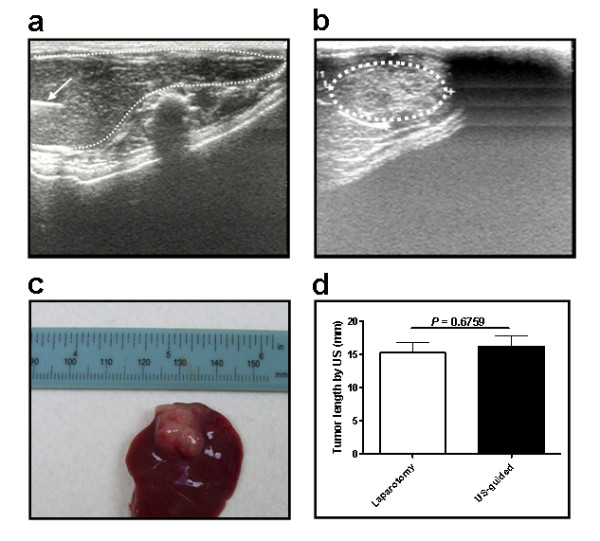
**Ultrasound (US) -guided implantation of orthotopic HCC**. (a) US-guided implantation on day 1. The needle track was shown on the left of the picture (arrow). (b) US follow-up showing tumor growth on day 10. (c) The appearance of HCC after the sacrifice of rats. (d) No significant difference in sizes of tumor formation with regard to the methods of induction by US and laparotomy were found (Number of rats: US vs. laparotomy: 55 vs. 39, *P *= 0.6759).

To generate orthotopic HCC in immune-competent rats, N1-S1 cells were implanted into the liver lobes of the anesthetized SD rats under the guidance of high resolution US on day 1 (Figure 1a). After 10 days, US revealed prominent HCC in 60.4% (55/91) of animals with an average size of 16.23 mm (Figure 1b). The presence of HCC was further confirmed after sacrificing animals for caliper measurement (Figure 1c). The rate of tumor induction by US was comparable to that of laparotomy (55/91, 60.4% vs. 39/52, 75%) and no significant difference in the sizes of tumor was found between the two groups (Number of rats: US vs. laparotomy: 55 vs. 39, *P *= 0.6759, Figure 1d). Indeed, it took much less time to perform ultrasound implantation. Besides, the animals recovered better and were free of adhesive tumor nodules at injection sites. Therefore, ultrasound-guided implantation of N1-S1 cells into liver lobes is an effective means of inducing orthotopic HCC in SD rats.

### The ultrasound-measured HCC size highly correlated with the caliper measurement, plasma GOT level, and CT-measured tumor size

**Figure 2:**
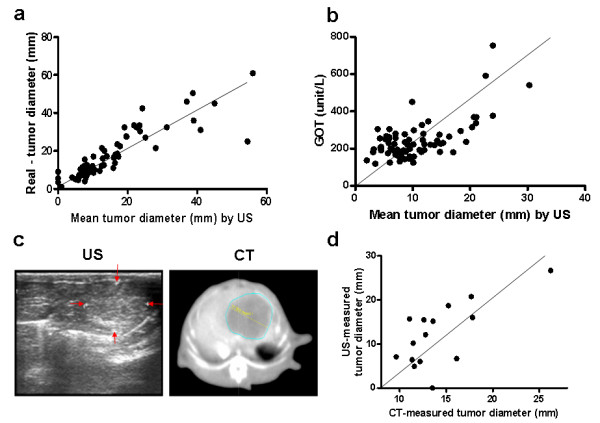
**Correlation of tumor sizes measured by ultrasound with various methods**. (a) Correlation of tumor sizes measured by US and calipers. (Pearson's correlation coefficient = 0.8697, n = 55, *** *P *< 0.0001). (b) High correlation between tumor sizes (US) and GOT level (Pearson's correlation coefficient = 0.6484, n = 71, *** *P *< 0.0001) was noted. (c) Photographs of liver tumors measured by US and CT scan. (d) High correlation of tumor measurements were obtained by US and CT scan (Pearson's correlation coefficient = 0.687, n = 15, * *P *< 0.05).

To evaluate the accuracy of US measurement, we first compared the US-measured HCC sizes with the caliper measurement immediately after sacrificing the animals. Arithmetic mean of tumor diameters were calculated from width and length or alternatively, tumor volume was estimated by the formula: volume = (shortest diameter)^2 ^× (longest diameter) × 0.5. By using arithmetic mean, the HCC sizes measured by US (16.23 ± 12.58 mm; n = 55) were similar to those of caliper measurement (18.63 ± 12.95 mm; n = 55). Moreover, there was an excellent correlation between US-measured and caliper-measured HCC sizes (Pearson's correlation coefficient = 0.8697, n = 55, *** *P *< 0.0001, Figure 2a).

The biochemical parameters in blood samples from HCC-bearing rats were determined to investigate the relationship between US-measured HCC sizes and plasma GOT/GPT levels. Interestingly, the US-measured HCC sizes significantly correlated with plasma GOT level (Pearson's correlation coefficient = 0.6484, n = 71, *** *P *< 0.0001; Figure 2b), but not with GPT (data not shown).

As CT scan is a fast and reliable means for acquiring two-dimensional tumor images, a portion of HCC-bearing rats were subjected to CT scan examination immediately after US diagnosis (Figure 2c). There was a significant correlation in the tumor size measured (arithmetic mean) by US and CT scan (Pearson's correlation coefficient = 0.687, n = 15, * *P *< 0.05; Figure 2d).

In addition, similar results were obtained by comparing the tumor volumes (US vs. caliper: n = 55, correlation coefficient= 0.7988, ***P < 0.0001; US vs. CT: n = 15, correlation coefficient= 0.9099, ***P < 0.0001). These findings strongly supported the fidelity of US-based monitoring of HCC progression in rats.

### Short-term and low-dose epirubicin chemotherapy led to stable disease in rats bearing established HCC

**Figure 3:**
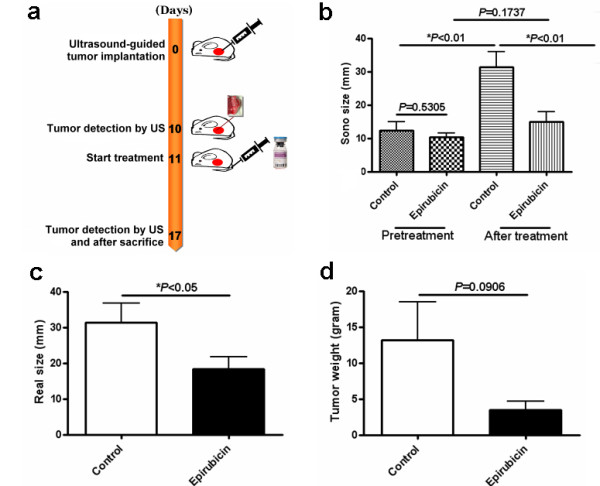
**Effects of short-term and low-dose epirubicin chemotherapy in rats with orthotopic HCC**. (a) Treatment scheme of short-term (7-day) and low-dose epirubicin for orthotopic HCC in the SD rats. (b) Mean tumor sizes before and after treatment in the control (n = 11) and epirubicin (n = 11) groups showed significant increase in sizes in the control group (* *P *< 0.01), but not in the epirubicin group (*P = 0.1737*). Besides, mean tumor size of the control group was significantly larger than that of the epirubicin group after the treatment (* *P *< 0.01) while the sizes of tumors were similar before treatment (*P = 0.5305*). (c) Significant difference was seen in the mean dissected (real) tumor sizes between the control and epirubicin groups after sacrifice (* *P *< 0.05). (d) No significant difference was noted in tumor weights between the control and epirubicin groups after sacrifice. (*P *= 0.0906).

**Figure 4:**
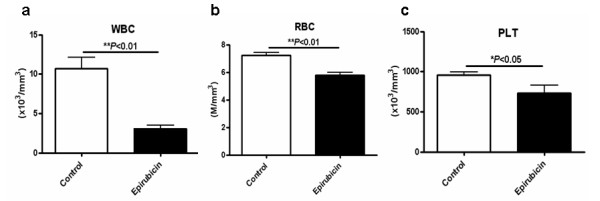
**Bone marrow suppression resulting from short-term and low-dose epirubicin chemotherapy was expressed by differences in peripheral blood cells between the two groups**. (a) Differences in the WBC (** P < 0.01), (b) the RBC (* *P *< 0.01) and (c) the platelet (* *P *< 0.05) counts between the treatment group (n = 11) and the control (n = 11) group.

Subsequently, rats bearing established HCC were subjected to short-term (7-day) and low-dose epirubicin therapy to validate this animal model for drug screening (Figure 3a). When tumors were successfully established on day 10, the animals were divided into the treatment (7-day low-dose epirubicin) group and the control group. After completion of treatment, ultrasound monitoring revealed that the tumor burden in the control group continued to increase (**P < 0.01; Figure 3b), whereas the tumors in rats treated with epirubicin showed no significant increment (*P *= 0.1737; Figure 3b). After sacrificing the animals, it was found that the tumor sizes of the control group were significantly larger than those of the epirubicin group (* *P *< 0.05; Figure 3c) while the tumor weights exhibited a similar trend but showed no statistical significance (*P *= 0.0906; Figure 3d). On the other hand, epirubicin treatment caused significant bone marrow suppression relative to the control group in terms of pancytopenia (Figures 4a, 4b and 4c).

### Short-term and low-dose epirubicin chemotherapy induced apoptosis and neovascularization blockade in HCC

**Figure 5:**
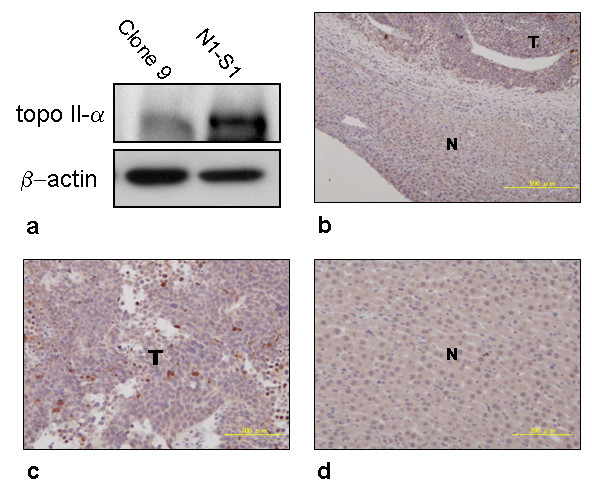
**Topoisomerase II-α (TOP2A) expression in N1-S1 HCC**. (a) Protein extracts isolated from N1-S1 HCC cells and normal hepatocytes (Clone 9) were subjected to the western blot analysis using TOP2A antibodies (1: 1000 dilution). As an internal control, the β-actin level was also determined. (b) Nuclear and cytoplasm TOP2A expressions in tumor (T) and non-tumor (N) specimens from rat HCC were shown. Original magnification ×100, the insets were then further magnified (×200) as shown in (c) tumor and (d) non-tumor parts.

**Figure 6:**
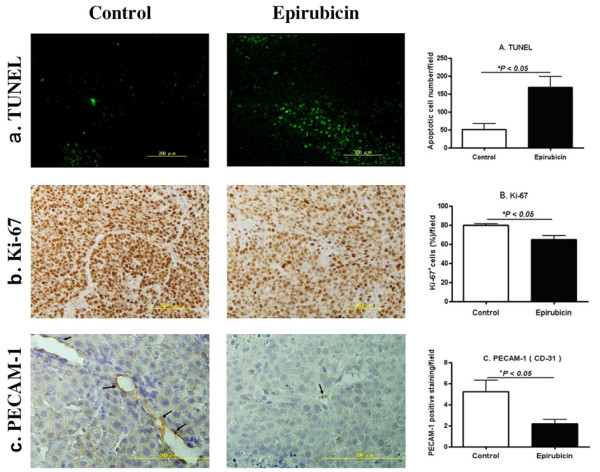
**Histological changes of HCC after short-term and low-dose epirubicin chemotherapy, control (n = 6) vs. epirubicin (n = 6)**. (a) Fluorescein-mediated TUNEL assay was performed to identify apoptotic nuclei (*green*), * *P *= 0.006. (b) Immunohistochemical staining of HCC by Ki-67 (*brown*) was done for examining the effect of epirubicin on the cell proliferation of HCC, * *P *< 0.0063. (c) Effect of short-term and low-dose epirubicin chemotherapy on tumor angiogenesis, arrows stand for the PECAM-1-positive blood vessels (* *P *= 0.0282).

Western blotting demonstrated abundant TOP2A enzyme expressions in N1-S1 cells using protein extracts from cancer cells and normal Clone 9 hepatocytes (Figure 5a). Consistent results were also obtained from immunohistochemical studies in tumor specimens (T: tumor vs. N: non-tumor parts; Figures 5b, 5c and 5d). To investigate the tumor-suppressing mechanism underlying epirubicin therapy, HCC samples were subjected to various histological analyses. TUNEL staining revealed a significant increase in the number of apoptotic cells in epirubicin-treated tumors as compared with the control group (Figure 6a). On the other hand, there was a significant reduction of Ki67-positive proliferating cells in the epirubicin-treated HCC (Figure 6b). Interestingly, the number of PECAM-1-positive blood vessels was also decreased in epirubicin-treated HCC (Figure 6c). Therefore, short-term and low-dose epirubicin chemotherapy had perturbed HCC proliferation through apoptosis induction as well as angiogenesis inhibition. Moreover, no significant correlation was obtained between the perfusion data (CT scan) and microvessel density changes (Figure 6c).

## Discussion

For the first time, the current study has demonstrated the feasibility of ultrasound-guided implantation of tumor cells into the native liver to create orthotopic HCC in immune-competent rodent. Compared with other HCC models including laparotomy, it possesses the advantages of easy manipulation as it took less than two hours to accomplish the implantation procedure in twenty animals, minimal trauma to the experimental animals, high reproducibility, early disease onset as reflected in the early detection of tumor by US within 10 days after implantation, and the ability to evaluate the effects of drug therapy on the tumors. Moreover, the results of tumor measurement by US were consistent with those assessed using functional CT scan and the actual sizes measured after sacrificing the animals, suggesting that US is an accurate and reliable means of size assessment of the induced tumors.

There are limited chemotherapeutic options for treatment of advanced HCC. Although the efficacies of many chemotherapeutic agents have been tested in treating HCC, the response rate is disappointing and ranges between 10% and 15%. Besides, no survival advantage has been demonstrated [21]. This may be due to the unbearable side effects of the conventional maximum tolerated dose (MTD) of chemotherapy upon patients who already have chronic liver diseases and cirrhosis, and the diversity in drug sensitivity due to genetic variations among the cancer cells [22]. In addition, tumor organization further diminishes chemotherapeutic efficacy through the development of multi-cellular drug resistance [23]. On the other hand, the method of metronomic chemotherapy is believed to attack the vascular system of the tumor instead of tumor parenchyma [14,15]. It has been demonstrated that this anti-angiogenic property can facilitate the cytotoxic effects of chemotherapeutic agents on the cancer cells [24-26]. This regimen can theoretically improve the tolerance of patients and prolong the treatment period, thereby enhancing the therapeutic efficacy of the cytotoxic drugs on the tumor cells.

Evidence from both basal and clinical researches has demonstrated the therapeutic effects of metronomic chemotherapy on certain kinds of solid tumors such as ovarian [27] and breast cancers. Colleoni *et al. *[28] have shown in their clinical trial that low-dose metronomic cyclophosphamide plus methotrexate is effective in the treatment of metastatic breast cancer with cheaper cost and prolonged tolerance to treatment. However, similar reports on the treatment of HCC are rarely found in the literature.

TOP2A over-expression seems to be correlating with the aggressiveness of HCC in terms of early age onset, advanced histological grading, microvascular invasion, chemoresistence, tumor recurrence and mortality [29,30]. These facts highlight the potential therapeutic value of epirubicin in targeting TOP2A in the management of HCC. Besides, epirubicin is one of the most frequently used chemotherapeutic agents in the treatment of HCC, either alone or in combination with other cytotoxic drugs, through various routes of administration including TACE (transcatheter chemoembolization), systemic chemotherapy, and trans-catheter arterial infusion chemotherapy [31-37].

Although the present study is only a short-term investigation that fails to elucidate the long-term therapeutic impact of epirubicin on HCC in an orthotopic animal model, several findings are noteworthy. Firstly, we found over-expression of TOP2A in N1-S1 HCC both in the protein and the histology levels. Secondly, we found a significant effect of the current regimen of epirubicin chemotherapy on HCC growth in this brief animal trial despite the association of bone marrow suppression. Thirdly, the short-term and low-dose epirubicin had high therapeutic potential in terms of histological changes of HCC. It significantly suppressed the proliferation of HCC cells as shown by the Ki-67 staining in comparison with the control group. In addition, TUNEL revealed an increase in the extent of apoptosis of HCC after chemotherapy. Furthermore, and perhaps most importantly, angiogenesis of the tumor endothelium decreased in the treatment group. However, the optimal dosages of metronomic chemotherapy, either chemopreventive or therapeutic, remain elusive [38]. Moreover, our orthotopic HCC animal model was not without shortcomings such as a lack of concurrent chronic liver diseases or cirrhosis that could not completely mimic the clinical condition.

Furthermore, Tang *et al*., [39] had recently reported an orthotopic advanced HCC model in which significantly improved overall survival was observed using various combinations of metronomic chemotherapy regimens with targeted anti-angiogenic drugs. Through implantation of tumor cells into the native liver through laparotomy, they successfully created orthotopic HCC in SCID mice. The size of the tumor was then monitored by using a novel non-invasive approach (transplantation of tumor cells that have been transfected with the β-hCG gene). The results of that study are impressive and encouraging. Both that study and our present study have successfully established valuable orthotopic HCC models that can be followed noninvasively either using a gene transfection method or sonographically. Our method has the additional advantage of being close to the clinical situation in which ultrasound is the major tool for following tumor progression. Also, both studies have demonstrated the potential of low-dose chemotherapy, either used alone or in combination with anti-angiogenic agents, in the treatment of HCC.

## Conclusions

In this study, encouraging result has been obtained by the ultrasound-guided tumor implantation of N1-S1 cells that has led to the growth of orthotopic HCC in about 60% of SD rats, which is comparable to the method of laparotomy. It is a fairly effective and feasible method for establishing an animal model of HCC for future therapeutic trials. It greatly reduces the time period expected for the tumor response to the experimental drugs. Moreover, we have shown the therapeutic efficacies of low-dose epirubicin on HCC growth in terms of tumor size, cancer cell proliferation, apoptosis as well as angiogenesis. On the other hand, the optimal dose of metronomic epirubicin should be substantiated by modifications of the current orthotopic animal model and running clinical trials in the future.

## Competing interests

The authors declare that they have no competing interests.

## Authors' contributions

HHC, MHT, JTC, HBP and KHL designed the study and analyzed the data. HHC, THH and MHT were responsible for writing the manuscript and revising it critically for important intellectual content. CKS performed the surgery in animals. HHC, THC HFC, EMW and HMK performed the immunoblot, biochemical, pathological and ultrasound experiments. All authors read and approved the final manuscript.

## Pre-publication history

The pre-publication history for this paper can be accessed here:

http://www.biomedcentral.com/1471-230X/10/83/prepub

## Supplementary Material

Additional file 1 **Affidavit of approval of animal use protocol**. The animal use protocol 'Development of novel diagnostic and therapeutic strategies for hepatocellular carcinoma' has been reviewed and approved by the institutional animal care and use committee (IACUC) of Kaohsiung Veterans General Hospital. Click here for file
